# Artificial Intelligence-Enabled Electrocardiography in Kidney Transplantation: Are We Ready to Rethink the Electrocardiogram?

**DOI:** 10.34067/KID.0000001170

**Published:** 2026-05-28

**Authors:** Laith Alzyood, Maria Ajaimy

**Affiliations:** Department of Medicine, Albert Einstein College of Medicine/Montefiore Medical Center, Bronx, New York

**Keywords:** cardiovascular events, clinical nephrology, kidney transplantation, biomarkers, epidemiological methods

Over the past decade, artificial intelligence (AI) has been transforming modern medical practice. From early medical decision-support tools to accelerated diagnostic evaluation, AI has the potential to narrow the longstanding gap between kidney and cardiovascular health by integrating complex, high-dimensional biomarkers into cardiovascular risk assessment in kidney disease—an area where traditional models often fall short.^1^ By applying pattern recognition algorithms on routine clinical data, AI can provide early detection, improve risk stratification, and enhance monitoring of treatment response. Among cardiovascular applications, AI-enabled electrocardiography (AI-ECG) has gained traction for predicting atrial fibrillation (AF). Using machine learning (ML) algorithms trained on sinus rhythm electrocardiography (ECG), AI-ECG has demonstrated the ability to identify patients at high risk of developing AF years before the first documented AF episode.^2^ AI-ECG therefore is a leading digital biomarker for preclinical AF, setting the stage for evaluating its role in kidney transplant recipients, a population at high-risk for AF-associated mortality, graft loss, and cardiovascular events.^3^

In this issue of *Kidney360*, Scalia *et al*.^4^ evaluated the utility of a pretransplant AI-ECG for predicting incident AF, mortality, and overall allograft survival among kidney transplant recipients. Using a retrospective cohort of 6246 adults transplanted at three tertiary transplant centers in the United States, the investigators applied AI-ECG risk tool—a previously validated risk tool with an area under curve of 0.87—to predict incident AF throughout a median follow-up of 7 years.^4^ Participants were stratified using an AI-ECG score cutoff of 5%, with 58.5% categorized as low-risk (score <5%) and 41.5% as high-risk (score ≥5%) for developing incident AF (Figure 1). Participants in the high-risk group were more likely to be men, older, and of non-White race and had a higher burden of comorbidities, including diabetes mellitus, coronary artery disease, and hypertension. They were also more likely to have been on dialysis before transplantation and to experience delayed graft function. Using an AI-ECG score cutoff of 5%, the AI-ECG score achieved higher sensitivity (72%) at the expense of lower specificity (62%) compared with the previously validated general population cutoff of 11% (sensitivity 56%, specificity 77%). High-risk scores were significantly associated with incident AF at 30 days (adjusted hazard ratio [aHR], 2.89; 95% confidence interval [CI], 2.05 to 4.09), 3 years (aHR, 2.54; 95% CI, 1.99 to 3.26), and 5 years post-transplant (aHR, 2.48; 95% CI, 1.99 to 3.09). Although 35.4% of incident AF events occurred within the first 30 days post-transplant, most events occurred beyond the acute peritransplant period. Importantly, exclusion of early events in a prespecified 30-day landmark analysis demonstrated that high-risk AI-ECG scores remained strongly associated with long-term incident AF, supporting the durability of AI-ECG–based risk stratification beyond transient perioperative hemodynamic and metabolic shifts. Beyond incident AF prediction, high-risk scores also were significantly associated with increased risk of mortality (aHR, 1.56; 95% CI, 1.30 to 1.88) and overall allograft failure (aHR, 1.50; 95% CI, 1.30 to 1.75) at 5 years postkidney transplantation. Subgroup analysis showed largely consistent associations between AI-ECG and outcomes, except for those with limited sample sizes, such as participants with reduced ejection fraction (<50%). This study clearly highlights vital deficiencies in arrhythmia diagnosis in patients with kidney disease and especially transplant recipients; however, it leaves us with several questions that cannot be answered, largely because of limitations inherent to the study's design.

**Figure 1:**
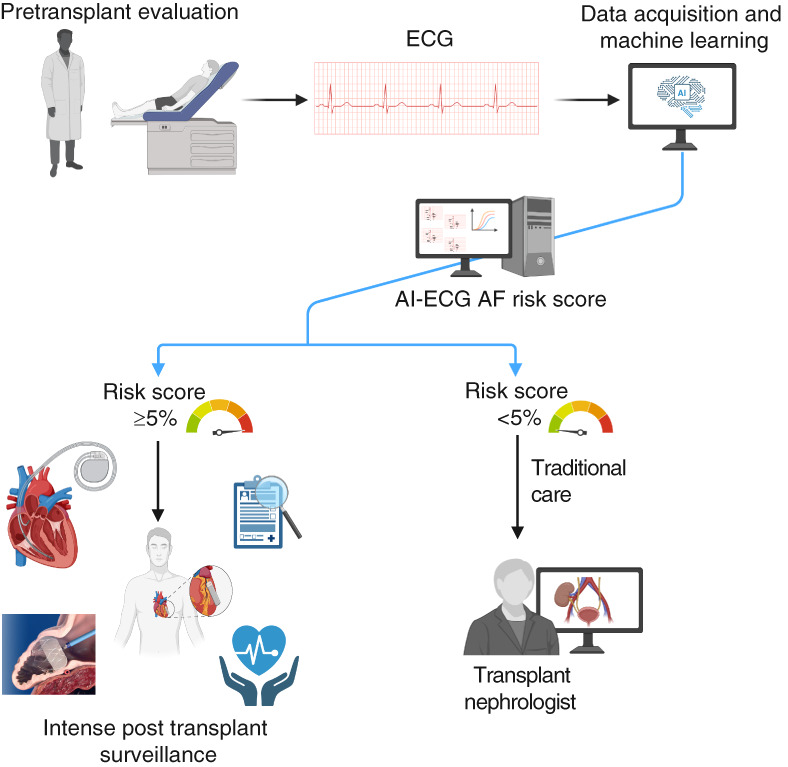
**Workflow for pretransplant AI-ECG risk assessment.** AF, atrial fibrillation; AI-ECG, AI-enabled electrocardiography; ECG, electrocardiography.

First, the retrospective design of the study and the predominantly White cohort raise concerns regarding selection bias and limit generalizability. Large population-based studies demonstrated that age-adjusted and sex-adjusted prevalence of AF is higher in White patients compared with Black and Hispanic patients, despite lower prevalence of traditional risk factors such as hypertension, obesity, and diabetes mellitus.^5^ This paradox may reflect true variation in AF susceptibility, or differences in health care access, and underscores the need for caution when interpreting findings from predominantly White cohorts to other racial and ethnic groups. Second, several clinically relevant covariates were unavailable in this analysis—including dialysis modality, ECG timing relative to dialysis cycle, hemodynamic instability, fluctuations in volume status, electrolyte abnormalities, infectious complications, immunosuppressive regimens, and rejection episodes—all of which are associated with increased AF risk, mortality, and allograft failure. As the authors appropriately noted, dialysis modality is no longer collected within the United Network for Organ Sharing, and the timing of ECG acquisition relative to dialysis could not be ascertained because ECGs were performed independently of dialysis sessions and were not systematically captured in national registries. The lack of information on dialysis modality and ECG timing represents a significant methodological limitation because prior studies demonstrated that AF risk is higher in hemodialysis than in peritoneal dialysis, and AF onset varies substantially across the dialysis cycle, peaking during dialysis and in the immediate postdialytic period.^6,7^ The lack of these covariates may introduce residual confounding into AI-ECG–based AF risk prediction, even when ML models are trained on sinus rhythm ECGs. Third, ECGs were not serially obtained. Choi *et al*., demonstrated that ML model trained on serial ECGs outperform ML model on the basis of a single ECG.^8^ The lack of serial ECG data may have reduced the predictive performance of the ML model used in this study. Finally, the AI-ECG model applied in this study was originally derived and validated in a general population cohort. Prior external validation studies, such as the work by Yuan *et al*. in the Veterans Affairs health system, have demonstrated preserved performance of AI-ECG for AF prediction when applied in different cohorts^9^; however, the cohorts were not representative of medical complexity of transplant recipients. Evidence suggests that AI-ECG algorithms can exhibit lower performance when applied to demographically or clinically distinct populations,^10^ reinforcing the need for caution when extrapolating performance to heterogeneous transplant recipients. Notwithstanding the limitations, this work by Scalia *et al*.^4^ has important clinical and research implications. Well-designed prospective studies, with appropriately matched cohorts, are needed to examine the predictive performance of AI-ECG for incident AF in kidney transplant recipients and to determine whether serial ECG acquisition improves risk prediction compared with single pretransplant ECG. Such studies should also evaluate whether AI-ECG performance differs when ECG is obtained during dialysis or in the immediate postdialysis period—when physiologic stress and electrolyte shifts may be greatest—compared with recordings obtained during the interdialytic steady state. Ultimately, future studies using AI-ECG to risk-stratify transplant recipients may help transform post-transplant care and individualize perioperative management strategies—including beta-blocker therapy or modulation of perioperative volume shifts— ultimately optimizing medical care for vulnerable transplant recipient populations.

From a health care perspective, there is a growing interest in implementing AI to enhance early arrhythmia detection, risk stratification, treatment response monitoring, and outcome prediction. First step toward this goal is to develop ML models with generalizability across diverse patient populations and ethnic groups, minimizing algorithmic bias. Equally challenging will be the integration of ML models into electronic health records to allow automated ECG analysis and real-time medical-decision support. Once rigorously validated and implemented, AI will have the potential to transform clinical practice and improve patients’ survival.

As the transplant community continues to improve long-term patient survival, attention must increasingly shift beyond traditional risk stratification. Incorporating validated ML models to better identify and mitigate arrhythmia risk represents the next important step. Such strategies may ultimately reduce cardiovascular morbidity, preserve allograft function, and help transplant recipients achieve longer survival and freedom from dialysis.

## Supplementary Material
