# Supplementary material for: Artificial Intelligence-Enabled Electrocardiography in Kidney Transplantation: Are We Ready to Rethink the Electrocardiogram?
Source: Kidney360. 2026 May 28;7(5):935–7. doi: 10.34067/KID.0000001170 (PMC13229422; doi:10.34067/KID.0000001170)
Supplement: Supplementary file 1 [file kidney360-7-0935-s001.pdf]

ASN American Society of Nephrology JASN Journal of the American Society of Nephrology KIDNEY360 Accessing Our World From Every Angle CJASN Clinical Journal of the American Society of Nephrology

ASN Journal Disclosure Form

As per ASN journal policy, I have disclosed any financial relationships or commitments I have held in the past 36 months as included below. I have listed my Current Employer below to indicate there is a relationship requiring disclosure. If no relationship exists, my Current Employer is not listed.

M. Ajaimy reports the following:
Employer: Montefiore Medical Center

I understand that the information above will be published within the journal article, if accepted, and that failure to comply and/or to accurately and completely report the potential financial conflicts of interest could lead to the following: 1) Prior to publication, article rejection, or 2) Post-publication, sanctions ranging from, but not limited to, issuing a correction, reporting the inaccurate information to the authors' institution, banning authors from submitting work to ASN journals for varying lengths of time, and/or retraction of the published work.

Name: Maria Ajaimy
Manuscript ID: K360-2025-001512R1
Manuscript Title: AI-ECG in Kidney Transplantation: Are We Ready to Rethink the ECG?
Date of Completion: January 14, 2026
Disclosure Updated Date: January 14, 2026

ASN American Society of Nephrology | JASN Journal of the American Society of Nephrology | KIDNEY360 Accessing Our World From Every Angle | CJASN Clinical Journal of the American Society of Nephrology

ASN Journal Disclosure Form

As per ASN journal policy, I have disclosed any financial relationships or commitments I have held in the past 36 months as included below. I have listed my Current Employer below to indicate there is a relationship requiring disclosure. If no relationship exists, my Current Employer is not listed.

L. Alzyood reports the following:
Employer: Montefiore Medical Center; and Honoraria: Sermo clinical surveys.

I understand that the information above will be published within the journal article, if accepted, and that failure to comply and/or to accurately and completely report the potential financial conflicts of interest could lead to the following: 1) Prior to publication, article rejection, or 2) Post-publication, sanctions ranging from, but not limited to, issuing a correction, reporting the inaccurate information to the authors' institution, banning authors from submitting work to ASN journals for varying lengths of time, and/or retraction of the published work.

Name: Laith Alzyood
Manuscript ID: K360-2025-001512R1
Manuscript Title: AI-ECG in Kidney Transplantation: Are We Ready to Rethink the ECG?
Date of Completion: January 13, 2026
Disclosure Updated Date: January 13, 2026
